# The incidence and health burden of earaches attributable to recreational swimming in natural waters: a prospective cohort study

**DOI:** 10.1186/1476-069X-12-67

**Published:** 2013-08-21

**Authors:** Timothy J Wade, Elizabeth A Sams, Michael J Beach, Sarah A Collier, Alfred P Dufour

**Affiliations:** 1 Office of Research and Development, United States Environmental Protection Agency, Chapel Hill, NC, USA; 2 Centers for Disease Control and Prevention, Atlanta, GA, USA; 3 Office of Research and Development, United States Environmental Protection Agency, Cincinnati, OH, USA

## Abstract

**Background:**

Earaches and outer ear infections are commonly associated with swimming. In this study, we estimated the excess risk and health burden of earaches due to swimming in natural fresh and marine waters using results from a survey of over 50,000 beachgoers at nine beaches across the United States.

**Methods:**

Prospective cohort studies were conducted at four freshwater and five marine sites in the United States and Puerto Rico. Beach visitors were enrolled on summer weekends and holidays. Ten to twelve days after the beach visit, respondents answered questions about health symptoms, including earaches or ear infections experienced since the beach visit. Economic and physical burdens were also obtained. Fixed slope, random intercept (beach site) multivariate logistic regression models were used to estimate the relationship between head immersion swimming exposure and earaches. Model results were used to calculate excess risk for earaches attributable to swimming.

**Results:**

The overall incidence of self-reported earache was 1.6% in the 10–12 days after the beach visit. Earaches were more frequent in head immersion swimmers compared to non-swimmers for all beach sites and age groups. Earaches were unassociated with water sample measures of fecal contamination and turbidity. After adjustment for covariates, we calculated 7.12 excess earaches among head immersion swimmers per 1,000 swimming events. Twenty-four percent of those with earache reported missing their regular activities; 28% visited a doctor; 4% visited the emergency room; and 31% and 40% used prescription and non-prescription medications, respectively.

**Conclusions:**

There are at least 128 million swimming events in natural waters annually. Such frequent exposures could result in 900,000 excess earaches, 260,000 visits to the doctor, 39,900 visits to the emergency room, nearly $4 million dollars in out-of-pocket expenditures on prescription and over-the-counter medications, and close to 75,000 hours of clinician time. More accurate estimates of swimming exposure are needed to improve population burden and associated cost estimates.

## Background

Ear pain (otalgia) is one of the most common reasons for visits to primary care physicians and medical care providers [1,2]. Causes include inner ear infections (otitis media), external ear infections (otitis externa), foreign bodies and trauma [1]. Otitis media is the most common cause of ear pain, occurs primarily in young children, peaks during the winter months and is associated with respiratory infections [3]. In contrast, otitis externa is regularly associated with swimming and diving (often called “swimmer’s ear”), [4-6], affects all ages [7-9] and peaks during the summer [7,10]. However, because outer ear infections can develop anywhere from hours to days after exposure, they may not always be accurately attributed to swimming or other water exposures [11]. Moisture, humidity and water in the ear canal are thought to remove the protective lining (cerumen, or ear wax) and increase the pH, increasing susceptibility to infection [8]. Among divers, otitis externa was associated with a change in the normal microbial flora from Gram-positive to Gram-negative species shortly after immersion [12]. *Pseudomonas aeruginosa*, *Staphylococcus epidermidis* and *Staphylococcus aureus* are the bacterial agents most frequently isolated from the ear canal of those diagnosed with otitis externa [10]. Some studies have linked the presence of *Pseudomonas aeruginosa* in waterbodies to cases of otitis externa in swimmers [13,14].

The health-care burden associated with earaches and ear infections is considerable. An estimated 2.4 million U.S. health-care visits (8.1 visits per 1,000 population) were associated with a diagnosis of acute otitis externa (ambulatory care or emergency room) in 2007 [15]. To develop estimates of the incidence and health burden of earache directly attributable to swimming in natural waters, we used data from over 50,000 beachgoers enrolled in the National Environmental and Epidemiological Assessment of Recreational Water Study (NEEAR Water Study) at nine beach sites (4 marine, 5 freshwater) [16-18].

## Methods

### Study sites, subject recruitment and enrollment

The data used for this analysis were obtained from interviews of beach goers at the nine beach sites studied as part of the NEEAR Water Study. The first phase focused on beaches in temperate climates, located near one or more treated sewage discharge points. Four freshwater beach sites located in the Great Lakes (Lake Michigan and Erie) were studied in 2003 and 2004 [16,17]. Three marine beach sites (Fairhope, Alabama; Edgewater, Mississippi; and Goddard Memorial State Park, Rhode Island) were studied in 2005 and 2007 [18]. In 2009, two additional beach sites were studied, a temperate marine beach site which was not known to be impacted by sewage discharge (Surfside, South Carolina) [19] and a tropical beach site (Boquerón, Puerto Rico) [19].

The study design, subject recruitment and questionnaire administration have been described previously [16-18]. Briefly, beach-goers were offered enrollment on summer weekends and holidays between approximately 11:00 AM and 5:00 PM. Respondents who had a household member of consenting age, were able to converse in English or Spanish, and had not participated in the study within the prior 28 days were eligible to enroll. The health survey was administered in three parts: enrollment, exit interview, and telephone interview. Interviewers approached beach-goers on weekends and holidays during the summer. An adult answered questions for other household members. The exit interview was conducted as they were leaving the beach for the day and included questions about demographics, swimming and other beach activities, consumption of raw or undercooked meat or runny eggs, chronic illnesses, allergies, acute health symptoms in the past 3 days, contact with sick persons in the past 48 hours, other swimming in the past week, and contact with unfamiliar animals. The telephone interview was conducted 10–12 days after the beach visit, and an adult answered questions for other household members who visited the beach. The telephone interview consisted of questions about health symptoms experienced since the beach visit and other swimming or water related activities, contact with animals, and consumption of high-risk foods since the beach visit. Economic and physical burdens experienced as a result of each illness were also obtained (for example, days missed from work, money spent on medications).

All study participants provided verbal consent. The study design, procedures and protocols were approved by the Institutional Review Boards for the Centers for Disease Control and Prevention and the University of North Carolina.

### Outcome definition

At the telephone interview, respondents were asked if they had “an earache, ear infection or runny ears” since the beach interview (referred to as “earache”). Participants reporting earache were also asked when the symptoms started and for how many days they lasted. They were also asked whether they or others lost time from work or regular activities, consulted a health care provider, visited an emergency room, were admitted to the hospital, and/or used prescription or over the counter medications as a result of the earache.

Participants who reported having an earache, ear infection or runny ears in the three days prior to the beach interview were considered prevalent cases and excluded from the analysis. Those who reported that their ear associated symptoms were due to allergies were also excluded.

### Swimming exposure

Because moisture in the ear is an important factor for swimming-related ear infections, head immersion or submersion in water was the primary exposure of interest. Three mutually exclusive exposure categories were generated: 1) Head immersion swimmers (“Swimmers”), were participants who reported submerging their head or putting their face in water; 2) Non-swimmers were those who reported no water contact; and 3) “Waders” were those who swam but did not immerse their head. Non-swimmers were used as the reference group for risk calculations.

### Data analysis

Earache (presence or absence) was the health outcome of interest and head immersion swimming was the primary exposure. Incidence of earache was tabulated and summarized by demographic characteristics, beach activities, and environmental exposures. Logistic regression models including a random intercept for beach site, were used to estimate the association between swimming and incidence of earache. Initial models included age category, race, sex, regular frequency of visits to the beach, miles traveled to the beach, burying body in sand, precipitation in the previous 17 hours, contact with unfamiliar animals, other swimming exposures, use of sunblock, use of insect repellent, use of ear plugs, presence of allergies, asthma, or chronic skin conditions and number of other beachgoers in the water. Final models were selected by sequentially removing variables from the full model (backward selection), minimizing Akaike’s Information Criterion (AIC) [20]. To assess whether combining age groups was appropriate, a model with multiplicative interaction terms between age category and swimming was compared with a model without these interaction terms using a Likelihood Ratio Test.

#### Burden of swimming-associated earache

Following final model selection, the excess risk (ER) associated with swimming, adjusted for covariates, was determined using “G-computation” [21] as described by Snowden et al. [22]. In this approach, the probability of earache for swimmers was estimated using coefficients from the selected logistic regression model and calculated as if all subjects had head immersion swimming exposure. The counterfactual probability of earache among non-swimmers was generated similarly, assuming all subjects were non-swimmers. The excess risk was then calculated as follows:

(1) $$\text{ER}(E)=\frac{1}{n}\overset{n}{\underset{i=1}{\sum }}P_{i}(E|S=1)-P_{i}(E|S=0)$$

where n is the total number of observations (subjects); *P*(*E*|*S* = 1) is the estimated probability of earache for head immersion swimming; *P*(*E*|*S* = 0) is the estimated probability of earache for no swimming exposure; and *ER*(*E*) is the excess risk of earache attributable to head immersion swimming. 95% confidence intervals were estimated by bootstrapping (1000 replications). *ER*(*E*) was expressed per 1000 swimming events.

To develop estimates of the impacts of earache on cost, personal time and health care utilization, the following responses were tabulated and summarized among those reporting earache: missing regular activities, others missing regular activities, phoning a doctor or nurse, visiting a doctor or nurse, visiting the emergency room, using prescription medicines, and using over-the-counter medications. The percentages of all respondents reporting these impacts were assumed to apply to the fraction of swimming-associated earache and were used as multipliers to attribute the health impact due to swimming:

(2) ER(E,I)=ER(E)×P(I|E=1)

where *P*(*I*|*E* = 1) is the proportion of respondents with earache reporting a particular health impact (e.g., missing work or school). *ER*(*E*,*I*) is the excess cases of swimming associated earaches resulting in the health impact.

## Results

### Incidence of earache

Survey data was collected on a total of 54,250 respondents. Excluding those with earache at baseline (N = 717), those with missing responses for earache (N = 176), and earaches due to allergy (N = 95), the overall incidence of earache was 1.6% (N = 829/53,262) in the 10–12 day follow up period. Descriptive and demographic characteristics for these subjects are shown in Table 1. Incidence was lowest among those over 50 years of age (1.0%) and highest among children 1 year and under (2.4%). To determine swimming associated risk, waders (those who entered the water but did not immerse their head) were also excluded, leaving 39,418 remaining. Among these subjects, 1.6% (N = 680/39,418) also reported earache. Numbers of swimmers and non swimmers by age group and beach site are shown in Table 2.

**Table 1:** **Beachgoers with and without earache 10–12 days after beach visit, by demographic characteristics**^**1,2**^

	**Earache**					
	**No**		**Yes**		**Total**	
	**No.**	**%**	**No.**	**%**	**No.**	**%**
*Age category*						
1 and under	1058	97.6	26	2.4	1084	100.0
2–5	3478	98.0	70	2.0	3548	100.0
6–10	4819	97.7	111	2.3	4930	100.0
11–19	7839	98.4	125	1.6	7964	100.0
20–50	26723	98.5	407	1.5	27130	100.0
Over 50	7586	99.0	80	1.0	7666	100.0
*Sex*						
Male	23289	98.6	333	1.4	23622	100.0
Female	29037	98.3	493	1.7	29530	100.0
*Race*						
Non-white	21060	98.4	337	1.6	21397	100.0
White	31241	98.5	489	1.5	31730	100.0
*Swimmingexposure*						
No contact	14037	99.0	144	1.0	14181	100.0
Contact withouthead immersion	13351	98.7	179	1.3	13530	100.0
Head immersion	24736	98.0	501	2.0	25237	100.0
*Contact withunfamiliar animals*						
No	48059	98.5	727	1.5	48786	100.0
Yes	4371	97.7	102	2.3	4473	100.0
*Used sunblock*						
No	19195	98.7	250	1.3	19445	100.0
Yes	32964	98.3	573	1.7	33537	100.0
*Used insect repellent*						
No	47355	98.5	723	1.5	48078	100.0
Yes	4824	97.9	101	2.1	4925	100.0
*Asthma*						
No	48615	98.5	733	1.5	49348	100.0
Yes	3786	97.5	96	2.5	3882	100.0
*Chronic skin condition*						
No	49942	98.5	780	1.5	50722	100.0
Yes	2454	98.0	49	2.0	2503	100.0
*Allergies*						
No	43808	98.6	635	1.4	44443	100.0
Yes	8569	97.8	194	2.2	8763	100.0
*Used earplugs*						
No	51781	98.4	816	1.6	52597	100.0
Yes	568	97.8	13	2.2	581	100.0
*Other swimming*^3^						
No	35708	98.8	449	1.2	36157	100.0
Yes	16648	97.8	379	2.2	17027	100.0
*Beach type*						
Fresh	20437	98.7	267	1.3	20704	100.0
Marine	31996	98.3	562	1.7	32558	100.0

^1^Including waders, excluding those with earache at baseline and earache due to allergy (N = 53,262).

^2^Categories may not add to 53,262 due to missing values.

^3^Other head immersion swimming in the 1-week prior to the beach visit.

**Table 2:** **Head immersion swimmers and non-swimmers, by beach site and age group**^**1,2**^

	**Head under water**					
	**No**		**Yes**		**Total**	
	**No.**	**%**	**No.**	**%**	**No.**	**%**
*Age category*						
1 and under	355	48.1	383	51.9	738	100.0
2–5	379	14.8	2185	85.2	2564	100.0
6–10	340	8.1	3864	91.9	4204	100.0
11–19	1448	21.8	5193	78.2	6641	100.0
20–50	8265	43.0	10970	57.0	19235	100.0
Over 50	3188	59.6	2158	40.4	5346	100.0
Total	13975	36.1	24753	63.9	38728	100.0
*Beach*						
Boquerón, PR	2915	22.8	9878	77.2	12793	100.0
Edgewater, AL	370	46.8	421	53.2	791	100.0
Fairhope, MI	834	56.8	634	43.2	1468	100.0
Goddard, RI	1558	66.9	772	33.1	2330	100.0
Huntington, OH	1513	74.9	506	25.1	2019	100.0
Surfside, SC	1722	21.5	6274	78.5	7996	100.0
Silver, MI	3324	46.1	3881	53.9	7205	100.0
West, IN	708	37.2	1197	62.8	1905	100.0
Washington Park, IN	1237	42.5	1674	57.5	2911	100.0
Total	14181	36.0	25237	64.0	39418	100.0

^1^Excluding waders, those with earache at baseline, earache due to allergy and those with missing data on earache at follow up.

^2^Categories for age do not add to 39,418 due to missing values.

**Figure 1:**
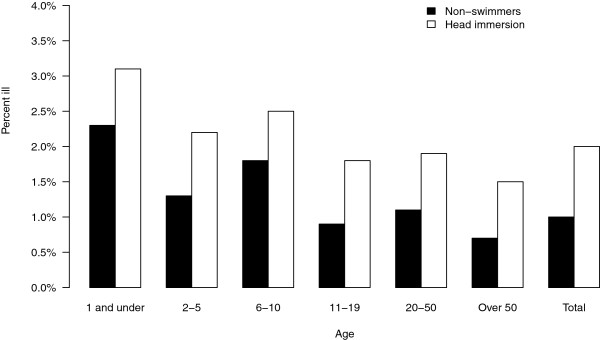
**Percent reporting earache by age group.** Percentage of non-swimmers and head immersion swimmers reporting earache at the follow up interview, 10–12 days after the beach visit, by age group.

**Figure 2:**
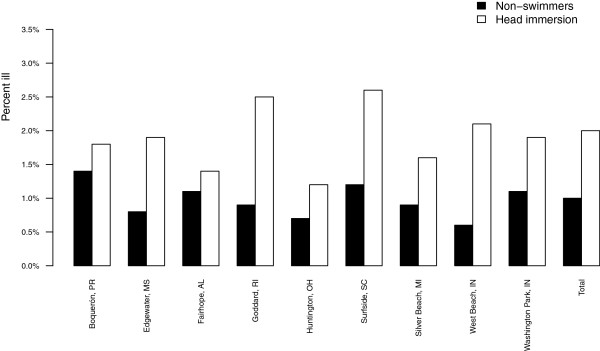
**Percent reporting earache by beach.** Percentage of non-swimmers and head immersion swimmers reporting earache at the follow up interview, 10–12 days after the beach visit, by beach site.

The incidence of earache for swimmers and non-swimmers by age category and beach site are shown in Figures 1 and 2. For all beaches and age groups combined, the unadjusted incidence of earache among swimmers was 2.0% (501/25,237) and 1.0% in non-swimmers (144/14,181). Earache incidence was elevated among swimmers at each beach site and across every age group. There was little difference in the incidence of earache across beach sites (Figure 2).

Earache incidence among swimmers was not associated with the level of fecal contamination as measured by *Enterococcus* using culture or quantitative polymerase chain reaction (qPCR) at freshwater and marine sites [17,18] or in the pooled data set (results not shown). Daily average turbidity measures were also unassociated with earache among swimmers (Adjusted Odds Ratio = 0.97, p = 0.86 per 10-fold increase).

Among swimmers, increasing time spent in water was not associated with an increase in the incidence of earache (p = 0.30). Those spending more than two hours reported earaches at the approximately same frequency as those spending 15 minutes or less in the water (2.0% vs 1.8%, respectively).

### Regression models and excess risk

Adjusted estimates from fixed slope random-intercept logistic regression models are shown in Table 3. Significantly elevated risks were observed for swimmers (AOR = 1.67; 95% CI: 1.35–2.07). Elevated odds ratios were also observed for very young children under 1, and this effect was more pronounced at freshwater sites. Other chronic health conditions, asthma and allergies were also associated with earaches. In contrast to head-immersion swimmers, waders did not have a statistically elevated incidence of earache compared to non-swimmers (AOR = 1.19; 95% CI = 0.95–1.49; p = 0.13; models not shown).

**Table 3:** Adjusted odds ratios for earache at marine and fresh water sites

	**All sites**		**Marine**		**Fresh**								
	**AOR**	**95% CI**	**AOR**	**95% CI**	**AOR**	**95% CI**							
Head immersion swimming	1.67****	1.35,2.07	1.56***	1.21,2.00	2.10****	1.48,2.99							
*Age* (ref. 20-50)													
Age 0–1	2.02**	1.28,3.21	1.55	0.84,2.88	3.00**	1.48,6.07							
Age 2–5	1.13	0.83,1.53	1.29	0.90,1.84	0.79	0.45,1.39							
Age 6–10	1.27*	1.00,1.61	1.41*	1.07,1.87	0.94	0.61,1.45							
Age 11–19	0.88	0.70,1.11	1.00	0.77,1.31	0.61*	0.39,0.96							
Over 60	0.76+	0.57,1.01	0.82	0.59,1.13	0.52+	0.25,1.07							
Non-white race	1.24	0.95,1.62	1.33**	1.08,1.63	1.11	0.73,1.68							
Female	1.29**	1.10,1.52	1.27*	1.06,1.54	1.35+	1.00,1.83							
Unfamiliar animal contact	1.57***	1.23,2.00	1.72***	1.28,2.31	1.31	0.86,2.00							
Other swimming^1^	1.60****	1.36,1.89	1.61****	1.33,1.96	1.58**	1.16,2.16							
Used insect repellent	1.41**	1.09,1.81	1.44**	1.11,1.88	1.21	0.53,2.77							
Allergies	1.61****	1.33,1.95	1.76****	1.41,2.21	1.31	0.91,1.88							
Asthma	1.46**	1.13,1.87	1.38*	1.03,1.86	1.71*	1.05,2.80							

+ p <0.1, ∗ p <0.05, ∗∗ p <0.01, ∗∗∗ p <0.001, ∗∗∗∗ p <0.0001.

AOR = Adjusted Odds Ratio, 95% CI = 95% Confidence Interval.

^1^Other head immersion swimming in the 1-week prior to the beach visit. Estimates from a logistic regression model with a random intercept for beach.

Because of the frequency of otitis media infections (which are not normally attributed to swimming) among infants and young children, we conducted additional analyses excluding children one year of age and under and five years of age and under. We also examined the association between earache and swimming only among children five and under. Results are provided in the supplement (See Additional file 1: Table S1). When children five and under were excluded, the association between swimming and earache was slightly stronger (AOR = 1.80; 95% CI: 1.43–2.27) compared the full model with all subjects (AOR = 1.67; 95% CI: 1.35–2.07, Table 3). Among children five and under association between head immersion swimming and earache was slightly attenuated compared to those over five (AOR = 1.52, 95% CI = 0.80–2.88), but the sample was relatively small (96 earaches reported).

Models with multiplicative interactions terms between swimming and beach site and age category did not improve the model fit as indicated by the AIC or Likelihood Ratio Test. Because of the consistency of the association between earache and swimming exposure across beaches and age groups, we used models that combined across these factors. We accounted for potential heterogeneity in the effect across beach sites with a random intercept term.

**Figure 3:**
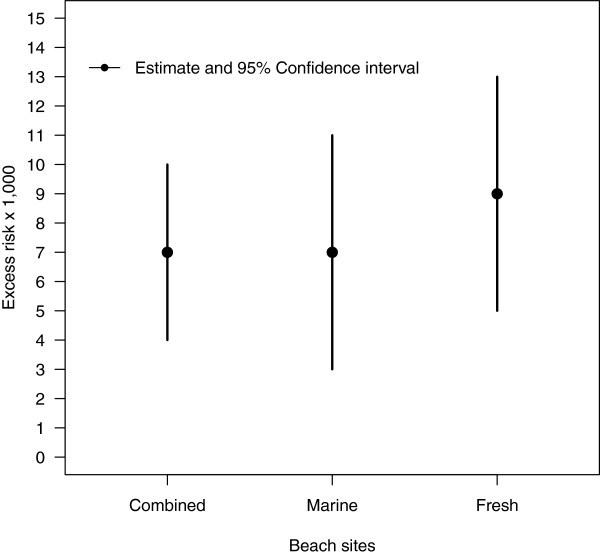
**Excess earaches per 1,000 swimming events.** Excess earaches per 1,000 swimming events among head immersion swimmers. Excess risk estimated from logistic regression models (see text for description).

Excess risks for earaches, *ER(E)*, estimated from the logistic regression models for marine, fresh and combined beach sites are shown in Figure 3. Across all beach sites, there were 7.12 excess earaches (95% CI: 4.1–10.2) for every 1000 swimming events. Separate estimates for freshwater and marine beach sites were similar to the combined estimate: 9.11 per 1000 (95% CI: 4.78–13.44) and 7.10 per 1000 (95% CI: 3.08–11.11), respectively (Figure 3).

### Health burden due to earaches

Among all subjects, 829 participants reported a new episode of earache (excluding those with earaches due to allergy and those reporting earache at baseline). Percentages of those with earaches who reported an economic or health care impact (*P(I|E = 1)*) are shown in Table 4. Twenty-four percent missed their regular activities, 31% used prescription medication and 41% used over-the-counter medications. Four percent visited the emergency room. Children one year of age and under and two to five years visited a doctor for earaches more frequently (58% and 54%, respectively) than older children and adults. The median duration of earache was three days, with a median of two days that impacted regular activity. Respondents who reported spending money on medications spent a median of $7 on prescription medications and $5 on non-prescription medications.

**Table 4:** Percentage of respondents with earache (N = 829) reporting missing regular activities, visiting the doctor or using medication

	**N**	**%**
Missed regular activities	201	24.3
Others missed regular activities	30	3.6
Phoned doctor or nurse	122	14.7
Visited doctor or nurse	235	28.3
Visited ER	36	4.3
Used prescription medication	257	31.0
Used over-the-counter medicine	337	40.6

Applying Equation 2, the earache-associated health impacts resulting from swimming exposure are shown in Table 5. The excess risk estimates for earaches resulting in medication are 2.89 and 2.21 per 1,000 swimming events (Table 5), resulting in swimming-associated earache medication costs of $15.47 (2.21 × $7) for prescription medications for every 1,000 swimming events and $14.45 (2.89 × $5)for non-prescription medications for every 1,000 swimming events.

**Table 5:** Earaches and earache-associated health impacts due to swimming

**Impact**	**Excess risk per 1000,*ER*(*E*,*I*)**
Number of earaches	7.12
*Earaches resulting in:*	
Missed regular activities	1.73
Phone doctor or nurse	1.05
Visit to doctor or nurse	2.02
Visit to emergency room	0.31
Use of over-the-counter medication	2.89
Use of prescription medication	2.21

## Discussion

Data collected from a survey of over 50,000 beachgoers were used to estimate the incidence and burden of earaches resulting from swimming in natural waters. For every 1000 swimming events in natural waters there were slightly more than 7 excess earaches. The effect we observed was relatively constant across beach sites and age groups. Although young children reported a higher overall incidence of earache, the risk associated with swimming exposure was slightly lower than other age groups. This is consistent with the age distribution of otitis externa which affects all age groups [7,8], and young children are not necessarily at higher risk. Earaches in young children and infants are often attributable to otitis media and very young children are also unlikely to experience extensive head-immersion swimming exposures.

The health care burden and economic impacts of swimming-associated earaches are substantial. The National Survey on Recreation and the Environment [23] estimated 41.3% (weighted to represent the United States population) of the population over 16 years of age had “Swam in a Lake, Ocean or Stream” during the past year. Assuming this percentage applies to the 2011 total United States population of 311,591,917 [24], the number of people who swim at least once per year in a natural waterbody is estimated as: 41.3% × 311,591,917 = 128,687,462. Applying the excess risk *ER*(*E*) estimate of 7.12 per 1,000 swimming events in natural waters results in 916,000 earaches due to these swimming exposures. Assuming the burden estimates we observed apply nationally, these earaches would result in 260,000 visits to the doctor, 39,900 visits to the emergency room and nearly $4 million dollars spent on prescription and over-the-counter medications due to earaches associated with swimming in natural waters. Since most people who swim likely do so numerous times each year (the NRSE only reported those who reported any swimming in lakes, oceans or streams annually), these are likely underestimates.

The costs for over-the-counter and prescription medications reported by participants are a fraction of the direct health care cost of earache. The average cost of a doctor’s office or emergency room visit for otitis externa (including out-of-pocket and insurer costs for the visit and prescription medication) has been estimated to be $200 [15]. Assuming these costs apply to earache, this implies $59,980,000 in direct health care costs to patients and insurers (260,000 office visits + 39,900 emergency room visits × $ 200). Additionally, each visit was estimated to occupy 15 minutes of clinician time, resulting in 74,975 hours of clinician time (299,900 visits × 0.25 hours/visit) spent on visits for earache attributable to swimming in natural waters. Roughly one-third of those visits could be expected to result in prescriptions for systemic antimicrobial medications, instead of recommended topical antimicrobials [25], equivalent to 98,967 antimicrobial prescriptions ( $299,900\times \frac{1}{3}$) attributable to swimming in natural waters. Such systemic antibiotic prescriptions may often be unnecessary in the absence of complicating conditions [25].

In order to estimate the health burden attributable to swimming-associated earache, we assumed that the health burden and impact of earache for all respondents were representative of the swimming-associated fraction. Although we are unaware of evidence to contradict this assumption, we could not directly evaluate it or confirm it with our data.

This analysis also does not consider swimming in pools, water parks and other chlorinated or treated venues. Although swimmer’s ear is known to be associated with these exposures [14], it is unclear whether the excess risk estimates of 7.12 earaches per 1000 swimming events for natural waters would be accurate for these conditions.

Our results are based on self-report of earache. Due to this limitation, we cannot exclude the possibility that at least some of the risk attributable to swimming exposure was a result of over-reporting of earache among swimmers. Swimmers and non-swimmers were unblinded with regard to the primary exposure of interest, head-immersion swimming, and as a result, it is possible that swimmers over-reported earaches based on this knowledge of exposure, resulting in an overestimate of the true excess risk of earache associated with swimming. However this bias, if present, may not have been strong. Subjects reported on numerous symptoms as part of the NEEAR Water study and earaches were not particularly emphasized in relation to these other symptoms. Swimming and non-swimming respondents were therefore probably unlikely to be abnormally or specifically focused on their earache symptoms. Unlike several other symptoms studied, earache was consistently elevated among swimmers relative to non-swimmers across beach sites and age groups. This consistency of effect was not observed for other non-enteric symptoms, and several showed little association with swimming following adjustment for covariates (respiratory, eye irritations) [17,18]. It seems unlikely that a reporting bias, if present, would specifically affect earache and not also affect other types of symptoms. The relatively constant association across age groups is consistent with otitis exerna in that age groups are affected approximately equally. Finally, “earache” is a relatively objective symptom with which most people are familiar with, and self-reported earache has been shown to agree well with medical records [26].

We did not clinically confirm or diagnose any of the self-reported earaches as otitis externa. It is likely that some of the excess earaches were due to trauma or other irritation so we cannot determine the excess risk specifically attributable to otitis externa. Nonetheless, preventative measures can be taken to reduce the risk of ear infections following swimming exposure. Clinical reports recommend the use of earplugs as a preventative measure was well as the use of over-the counter acidifying agents with alcohol or other astringent and drying the ears after swimming with a hair dryer on the lowest setting [4,8].

Earaches were associated with swimming, but not water quality as measured by the fecal indicator bacteria *Enterococcus* or turbidity. Swimmer density was also not an important determinant of earaches in our data (results not shown). It is possible that earaches were associated with a water quality parameter we did not measure, although consistent associations between otitis externa and water quality have not been established. Some previous studies have linked otitis externa among swimmers to the presence of *Pseudomonas aeruginosa* in ambient waters [13] and in pools [14], whereas others have not [27,28]. Swimming exposures may also result in conditions (e.g., moisture, humidity, inflammation, or trauma) where normal, endogenous flora of the ear canal can cause otitis externa [8,29]. Moisture from swimming or bathing is a known risk factor for otitis externa and can remove the protective layer of ear wax (cerumen), raise the pH and create conditions favorable to bacterial growth [8]. These changes may also cause itching in the external auditory canal, adding the potential for scratching and subsequent infection. Individual factors which we did not measure also increase susceptibility to otitis externa following swimming such as narrow or partially obstructed ear canals [11]. Duration of time in water, which has been noted as a risk factor for otitis externa in some studies [27] was unassociated with earache among swimmers in our study. Individual susceptibility or other measures of exposure intensity may outweigh the importance of duration in the water in the development of swimming-associated earache.

## Conclusion

In summary, from an analysis of over 39,000 beachgoers at nine freshwater and marine beach sites there were 7.12 excess earaches for every 1,000 head immersion swimming events. We further estimated that for every 1,000 swimming events there were 1.73 earaches that resulted in missed work or activity; 1 that resulted in a phone call to a doctor; 2 in visits to a doctor and 0.31 in visits to an emergency room. As there are over 128 million swimming events in natural waters annually, the population health burden attributable to swimming-associated earaches is considerable. Improved estimates of swimming in natural waters will provide a more accurate estimate of the health burden of swimming associated earaches and other health effects related to swimming exposures.

## Abbreviations

AOR: Adjusted odds ratio; ER: Excess risk; ER(E): Excess earaches associated with head immersion swimming; ER(E, I): Excess swimming-associated earaches resulting in a health impact; AIC: Akaike’s Information Criterion; qPCR: Quantitative polymerase chain reaction; CI: Confidence interval; p: p-value; EPA: United States Environmental Protection Agency; CDC: Centers for Disease Control and Prevention.

## Competing interests

The authors declare that they have no competing interests.

## Authors’ contributions

TW led data analysis, interpretation, and manuscript preparation. ES led data collection. MB developed the study design. SC provided calculations for health burden estimates. AD oversaw all aspects of the study. All authors read and approved the final manuscript.

## Supplementary Material

Additional file 1 Supplemental information. Click here for file
