# Supplementary material for: The incidence and health burden of earaches attributable to recreational swimming in natural waters: a prospective cohort study
Source: Environ Health. 2013 Aug 21;12:67. doi: 10.1186/1476-069X-12-67 (PMC3765573; doi:10.1186/1476-069X-12-67)
Supplement: Additional file 1 — Supplemental information. [file 1476-069X-12-67-S1.pdf]

Supplemental Information

Table S1: Adjusted odds ratios for earache for selected age groups. Estimates from a logistic regression model with a random intercept for beach.

	5 years and under		Over 1 year		Over 5 years	
	AOR	95% CI	AOR	95% CI	AOR	95% CI
Head immersion swimming	1.52	0.80,2.88	1.69****	1.36,2.11	1.80****	1.43,2.27
Age (ref. 20-50)[1]						
Age 0-1	1.53	0.88,2.66				
Age 2-5	1.00	1.00,1.00	1.13	0.84,1.54		
Age 6-10			1.27*	1.01,1.62	1.31*	1.03,1.67
Age 11-19			0.88	0.70,1.11	0.87	0.69,1.10
Over 60			0.76+	0.57,1.01	0.76+	0.57,1.03
Non-white race			1.24	0.94,1.64	1.23	0.93,1.64
Female			1.33***	1.13,1.56	1.35***	1.14,1.61
Unfamiliar animal contact			1.61***	1.26,2.05	1.58***	1.21,2.05
Other swimming[2]			1.59****	1.35,1.88	1.56****	1.30,1.86
Used insect repellant			1.42**	1.10,1.83	1.53**	1.16,2.01
Allergies			1.62****	1.34,1.97	1.69****	1.38,2.07
Asthma			1.48**	1.15,1.90	1.43*	1.08,1.88
Miles travelled[3]					0.92+	0.84,1.01
Rain (inches)[4]					1.22	0.95,1.56

+ p<0.1, * p<0.05, ** p<0.01, *** p<0.001, **** p<0.0001

AOR=Adjusted Odds Ratio, 95% CI=95% Confidence Interval

1: For Under Age 5 model reference group is Age 2-5

2: Other head immersion swimming in the 1-week prior to the beach visit

3: Miles travelled to the beach (0-20; 21-60; 61-100; > 100 miles)

4: Inches of precipitation in previous 17 hours
